# Supplementary material for: A Novel Subtype of Bovine Hepacivirus Identified in Ticks Reveals the Genetic Diversity and Evolution of Bovine Hepacivirus
Source: Viruses. 2021 Nov 2;13(11):2206. doi: 10.3390/v13112206 (PMC8623979; doi:10.3390/v13112206)
Supplement: Supplementary file 1 [file viruses-13-02206-s001.zip › viruses-1430830-supplementary1.pdf]

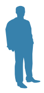

```
NC038882  CATCCTCTGAGCCCGCCCCTTCTGGC'''''''''TGCCCCCCCGACTCC'''''''''GACGTTGAGTC  7199
NC004102  CATCCTCTGAGCCCGCCCCTTCTGGC'''''''''TGCCCCCCCGACTCC'''''''''GACGTTGAGTC  7199
NC009823  CGGGC''''''GCTGCCGATTCCGGCAGTCAGACGCCTCCTGATGAGTTGGCCCTTTCGGAGACAGGTTC  7199
NC009824  CGACA-CACAGTCCAGCACTACTTCCAAGGTGCCCCCTTCTCCGGGAGGGGAG'''TCCGACTCAGAGTC  7199
NC009825  ---C---CGAGCTCAGACCGT---GATACAGACCTTACCACCCCAACT--GAGACCACAGAC-TCGGGCC  7117
NC009826  ------CCAGGATTCTGCGTT---GG-GTACTAGCAGCCAACACGAT--------TCGGGGC-CTGAGGA  7139
NC009827  ------CCGAGCTCCGGCCTC---GGAGGGTCAATAGCCGGCCCATCA-----TCTCCTGAC-CCGACAA  7153
NC030791  -------AGGAGGCACCCCCC---AGCGACTCCGCCATTAGTCTGGAT-----TCACCGGCGGCAAATGA  7130
```

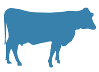

```
KP641125  CCTACGCCTGGGGCGGTCTTGAGCCT'''TTTCGTCAGCTCCAGATTTTTGACACTCCACCTCCTCCTTA  6365
KP641123  CCTACGCCTGGGGCGGCCTTGAGCCT'''TTCCGTCAGCTCCAGATTTTTGACACTCCACCTCCTCCTTA  6365
KP641127  CCTACGCCTGGGGCGGCCTTGAGCCT'''TTTCGTCAGCTTCAGATGTTTGACACTCCACCTCCTCCTTA  6365
KP641124  CCTACGCCTGGGGCGGCCTTGAGCCT'''TTCCGTCAGCTCCAAATGTTTGACACTCCACCTCCTCCTTA  6365
KP641126  CCTACGCCTGGGGCGGCCTTGAGCCT'''TTTCGTCAGCTCCAAATGTTTGACACTCCACCTCCTCCTTA  6365
MH027953  CCTATGCCTGGGGCGGTCTTGAGCCT'''TTCCGTCAGCTTGAAATGTTTGACACTCCACCTCCCCCTTA  6365
KP265950  '''''TTCTGGGCGGGGTTGCAGCCC'''TTCGGCCAATTACAGGTGTTTGAAACTCCACCACCCCCTTA  6365
KP265948  '''''TTCTGGGCAGGGCTGCAACCC'''TTCGGCCAATTACAGGTGTTTGAAACTCCACCACCCCCTTA  6365
KP265943  '''''TTCTGGGCGGGGCTGCAACCT'''TTTGGCCAACTACAGGTGTTTGAAACTCCACCACCCCCTTA  6365
KP265947  '''''TTTTGGGCGGGCCTGCAGCCT'''TTTGGACAATTACAGGTTTTTGAGACTCCACCCCCACCTTA  6365
KP265946  '''''TTTTGGGCGGGCCTGCAGCCT'''TTTGGACAATTACAGGTTTTTGAGACTCCACCCCCACCTTA  6365
MG781019  CCTACTTCTGGGGTGGACTTGAGCCC'''TTTCGCCAGTTGCAGATGTTTGACACTCCACCTCCACCATA  6365
MG781018  CCTATTTCTGGGGTGGACTTGAGCCC'''TTTCGCCAGTTGCAGATGTTTGACACTCCACCTCCACCTTA  6365
MG257793  '''''TTCTGGGCGGGGCTGCAGCCT'''TTCGGACAATTACAGGTATTTGACACTCCACCACCTCCTTA  6365
MG257794  '''''TTCTGGGCGGGGCTGCAGCCT'''TTCGGACAATTACAGGTATTTGACACTCCACCACCTCCTTA  6365
MH027948  '''''TTCTGGGCGGGTTTGCAGCCT'''TTTGGTCAACTCCAGGTGTTTGACACTCCACCTCCTCCTTA  6365
MN266283  CTTACTTCTGGGGCGGTCTCGAGCCC'''TTTCGTCAACTTCAGATGTTTGACACTCCACCCCCTCCTTA  6365
MN266285  CCTACTTCTGGGGCGGCCTTGAGCCT'''TTCCGCCAACTTCAGATGTTTGACACTCCACCTCCTCCTTA  6365
MN266284  CCTACTTCTGGGGCGGCCTTGAGCCT'''TTCCGCCAACTTCAGATGTTTGACACTCCACCTCCTCCTTA  6365
MZ221927  CCTACTTTTGGGGGGGGCTTGAACCT'''TTCCGCCAACTGCAGATGTTTGACACTCCACCTCCACCTTA  6365
MZ540979  CCTACTTTTGGGGGGGGCTTGAACCC'''TTCCGCCTACTGCAGATGTTTGACACTCCACCTCCACCTTA  6365
MZ540980  CCTACTTTTGGGGGGGGCTTGAACCC'''TTCCGCCTACTGCAGAAGTTTGACACTCCACCTCCACCTTA  6365
MN691105  '''''TTCTGGAGCGGTCTGACGCCGACGCTCGGTCAACTCCAAATCTTTGACACCCCGCCGCCACCGTA  6365
```

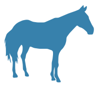

```
NC038425  TAACAATTACTTTAAACTTTGCTTGTTGCTTTTAGCTTATTCAGGCTTAGTTTCTTGT''''''''''''  1188
NC024889  TAATAATTACTTTAAATTATGCTTATTACTCTTGGCTTATTCTGGCTTAGTTTCTTGTACT'''''''''  1188
MT955624  AAATAATTACTTTAAGCTTTGCCTGTTGCTTTTAGCGTATTCTGGCTTAGTTTCTTGTGGGAACGATAAT  1188
MT955623  AAATAATTACTTTAAGCTTTGCCTGTTGCTTCTAGCGTATTCTGGCTTAGTTTCTTGT'''''''''GAT  1188
MT955622  AAATAATTACTTTAAGCTTTGCCTGTTGCTTCTAGCGTATTCTGGCTTAGTTTCTTGT'''''''''GAT  1188
MH027992  CAATAATTACTTTAAACTTTGCTTATTGCTTTTAGCTTATTCGGCATTAGTTTCTTGTGATGAT''''''  1188
MN734124  TAACAATTACTTTAAGCTTTGTCTGCTGCTTTTAGCTTACTCAGGCTTAGTTTCTTGTGAT'''''''''  1188
MK644936  TAACAATTATTTCAAGCTTTGCTTGCTACTTTTGGCATATTCTGGCTTGGTTTCTTGTGGCACCTCAGAA  1188
KX056116  TAACAATTACTTTAAACTTTGCTTATTGCTTTTAGCTTATTCAGGCTTAGTTTCTTGTAAT'''''''''  1188
KX421286  TAATAATTACTTTAAGCTTTGCCTATTGCTTTTGGCGTATTCTGGCTTAGTTTCTTGTGATTCT'''ACA  1188
KT880192  AAACAATTACTTCAAGCTGTGTTTACTGCTTTTAGCGTATTCTGCCTTGGTTTCCTGT'''''''''GAA  1188
```

Figure S1. Multiple sequence alignment showing the example of variation in genomics of hepaciviruses in bovine, humans, and equine.
