# Supplementary material for: A Novel Subtype of Bovine Hepacivirus Identified in Ticks Reveals the Genetic Diversity and Evolution of Bovine Hepacivirus
Source: Viruses. 2021 Nov 2;13(11):2206. doi: 10.3390/v13112206 (PMC8623979; doi:10.3390/v13112206)
Supplement: Supplementary file 1 [file viruses-13-02206-s001.zip › viruses-1430830-supplementary2.pdf]

Table S1. Information about the sequences used in this study.

**Virus species**	**GenBank accession number**	**Country**
Bovine hepacivirus	KP641125	Germany
	KP641123	Germany
	KP641127	Germany
	KP641124	Germany
	KP641126	Germany
	MH027953	Germany
	MH027948	Germany
	KP265950	Ghana
	KP265948	Ghana
	KP265943	Ghana
	KP265947	Ghana
	KP265946	Ghana
	MG781019	Brazil
	MG781018	Brazil
	MG257793	China
	MG257794	China
	MN266283	China
	MN266284	China
	MN266285	China
	MZ221927	China
	MZ540979	China
	MZ540980	China
	MN691105	China
Hepatitis C virus	EU781832	USA
	KC844047	China
	AM910652	Spain
	KF676352	China
	AF238486	Japan
	JX227965	United Kingdom
	KC844050	China

JX227953	United Kingdom
JX227968	Canada
JQ717260	India
LC414155	Japan
JX227954	United Kingdom
JX227955	United Kingdom
DQ418788	USA
KC844045	China
JX227963	United Kingdom
JX227958	United Kingdom
JX227961	United Kingdom
JX227970	United Kingdom
JX227979	United Kingdom
JX227962	United Kingdom
JX227960	United Kingdom
KC844046	China
NC009826	United Kingdom
DQ480524	China-Hong Kong
EF424629	Thailand
LC435027	Cambodia
DQ835764	Thailand
DQ314806	China-Hong Kong
DQ835770	Thailand
DQ835769	Thailand
DQ278893	China
EF424628	USA
DQ835767	Thailand
EF424627	Canada
EF424626	Canada
EF424625	Canada
LC435028	Cambodia
EU798761	China
NC030791	Canada

Equine hepacivirus	NC038425	USA
	NC024889	Japan
	MT955624	USA
	MT955623	USA
	MT955622	USA
	MH027992	Germany
	MN734124	China
	MK644936	China
	KX056116	South Korea
	KX421286	Germany
	KT880192	France
